# Mapping the vitiligo patient journey: from awareness to treatment or coping strategies

**DOI:** 10.3389/fresc.2024.1511053

**Published:** 2025-01-07

**Authors:** Y. Valle, T. Lotti, S. Towheed, J. Sigova

**Affiliations:** ^1^ Vitiligo Research Foundation, New York, NY, United States; ^2^ Department of Dermatology, University of Studies Guglielmo Marconi, Rome, Italy; ^3^ Michael G. DeGroote School of Medicine, McMaster University, Hamilton, ON, Canada; ^4^ Department of Neonatology, Faculty of Continued Medical Education of Pirogov Russian National Research Medical University, Moscow, Russia

**Keywords:** vitiligo, patient journey, quality of life, help-seeking behavior, self-care

## 1 Introduction

Dermatology encompasses over 3,000 distinct diseases, yet comprehensive data capturing the full patient journey—from onset to diagnosis and treatment—remains scarce (1). Traditional patient journey maps, where available, often depict linear progressions within healthcare settings, oversimplifying the experience. These models frequently fail to reflex the non-linear, and cyclical nature of chronic conditions like vitiligo.

Vitiligo, a chronic autoimmune disorder characterized by unpredictable skin depigmentation, affects approximately 1% of the global population, with prevalence ranging from 0.1% to 2.0% depending on the region (2). Despite recent advancements in the understanding and treatment of vitiligo, patients frequently encounter significant challenges in navigating their care journey (3). While clinical advancements have improved treatment options, a gap remains in understanding how patients experience and navigate their care journeys.

The Vitiligo Patient Journey Map (VPJM) was developed to bridge this gap. It offers a structured view of the patient's journey, highlighting critical touchpoints and decision-making moments that influence outcomes. Rooted in mixed-methods research, the VPJM aims to guide clinicians, empower patients, and inform health policy to create a more patient-centered approach to care.

## 2 Discussion

### 2.1 Methodology

The VPJM was developed using a rigorous mixed-methods approach (4). This included the analysis of 982 electronic patient records from a proprietary health record system, Vitiligo CloudBank (formerly Cloud MRM) (5), alongside an online survey of 532 patients.

The patient records, spanning 2012 to 2020, were accessed through the system's admin panel in January 2021. These de-identified records contained detailed data on vitiligo diagnoses, treatment specifics, and follow-up outcomes. Data extraction, facilitated by the system's built-in export feature into Excel, captured variables such as patient age, age at onset, body surface area (BSA) affected, Fitzpatrick skin type (I–VI), dates of updates, and types and numbers of treatments prescribed. Statistical analyses were performed using SAS 9.4 (SAS Institute Inc., Cary, NC, USA).

The survey, based on the previously published Vitiligo Questionnaire, Version 4.0, consisted of 40 questions across seven dimensions (6). Conducted from September 12 to November 20, 2021, it was emailed to over 21,500 potential participants worldwide. Respondents began with a brief screener to collect basic demographics, confirm a vitiligo diagnosis, and obtain consent. Of the 889 individuals who passed the screening, 532 completed the full 20-minute survey. No personally identifiable information was collected at any stage. Responses were coded and analyzed using NVivo for Mac (Lumivero, Denver, CO, USA).

To visualize the patient journey, a medium-fidelity digital wireframe was created using Miro, an online whiteboard tool (Los Angeles, CA, USA). The map was structured along an *X*-axis with three cardinal points—“White Spot,” “Beautiful,” and “Spotless”—representing phases of the journey characterized by distinct patient experiences and emotions. An initial *Y*-axis, intended to depict potential causes or triggers of vitiligo, was later omitted to streamline the design.

The prototype underwent multiple iterations, with input from a team of 11 experts and three dermatologists, experienced in treating vitiligo. Consensus was achieved on the interdependencies of each element before advancing the design.

This methodology allowed us to capture a wide range of patient experiences, from initial symptom recognition to long-term management strategies.

### 2.2 Key findings

Our analysis revealed consistent demographic patterns across both data sources. The median ages were 42 years (medical records) and 37 years (survey), with 51%–53% of participants being female in both groups. The median age at diagnosis was 28 and 32 years, with an average disease duration of 13 and 11 years, respectively. Additionally, 38%–32% of patients reported a known family history of vitiligo. The ethnic composition in the medical records was 3% Latino, 8% Asian, 9% Black, 69% White, and 11% multiracial, while the survey did not include ethnicity-specific questions.

Clinicians estimated a median body surface area (BSA) affected by vitiligo of 4.9%. Notably, 51.9% of patients had more than 5% BSA affected, likely reflecting a higher propensity for patients with more noticeable lesions to seek medical attention. Of all cases, 12% were minor or stable, requiring little to no therapeutic intervention and resulting in no significant sequelae.

A third of the patients had undergone an average of 6.2 different treatments over the course of managing their condition. These interventions included phototherapy (UVA, UVB, intense pulse light systems), topical and systemic treatments (all off-label), depigmentation and camouflage techniques, and various supplements (vitamins, minerals, plant extracts).

Disease progression varied significantly among the medical records: 6% of participants experienced no progression, 32% reported slow progression, while the majority faced medium to rapid progression. Over 40% of respondents had been initially misdiagnosed, with some variation in the rate across countries. Care was fragmented, with patients consulting an average of 2.3 specialists over 7.8 months—significantly shorter than the 2.4 years reported in the VALIANT study (7). Patients had also tried an average of 5.9 treatments in total. Diagnosis and treatment timelines varied geographically, ranging from days to several years.

Treatment adherence posed a significant challenge. While 45% of survey respondents reported taking weeklong treatment breaks, the actual rate of non-adherence is likely higher. The primary reason cited was a lack of noticeable improvement after 3 months. Some patients discontinued treatment prematurely, assuming their symptoms had resolved, while others struggled with barriers like social, educational, or domestic responsibilities, making regular phototherapy sessions difficult to sustain.

### 2.3 Map design

#### 2.3.1 Patient-friendly format and design

**Figure 1:**
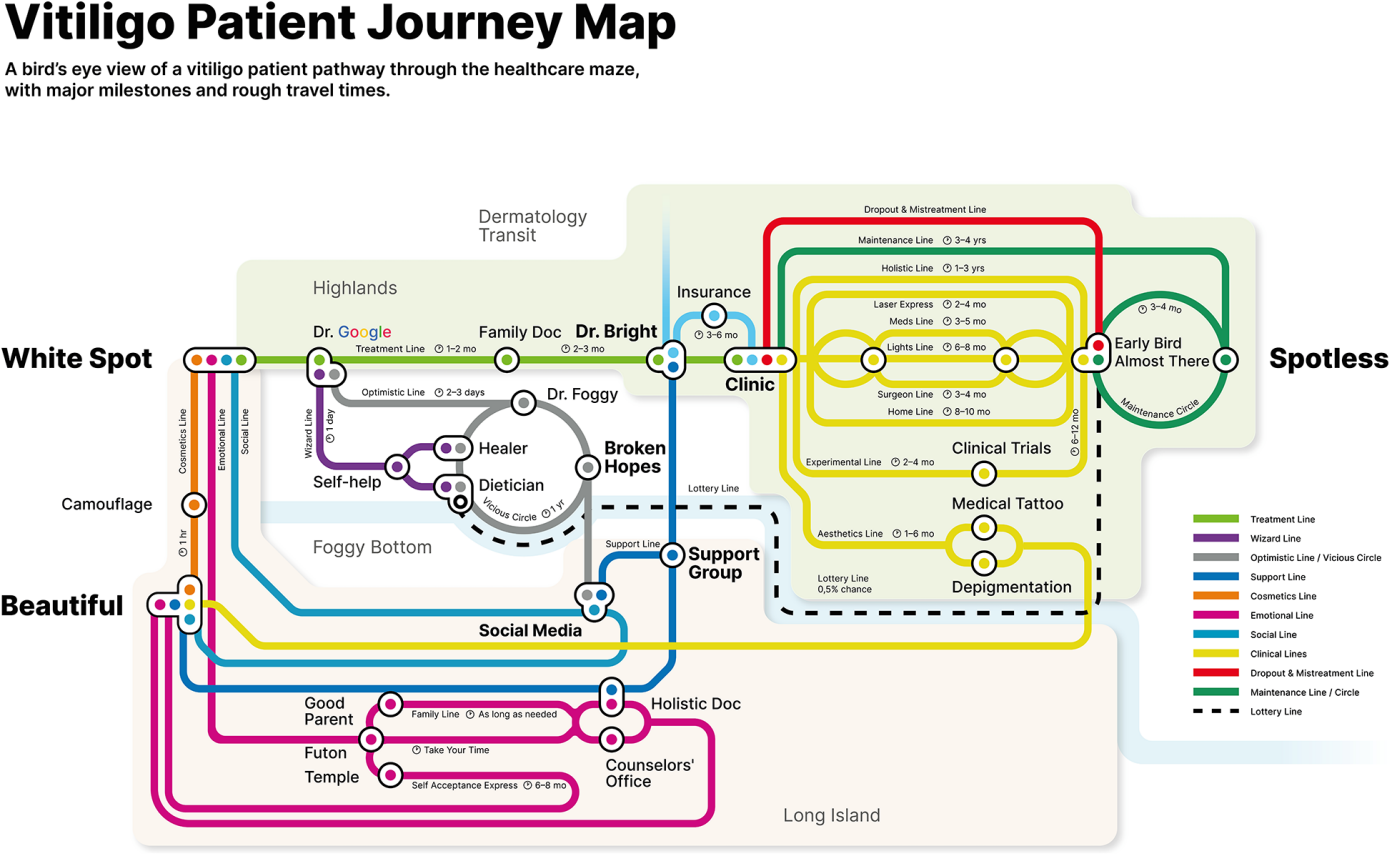
Vitiligo Patient Journey Map final design. A high-resolution version is available in multiple languages from the corresponding author.

The VPJM is designed to be intuitive and accessible for both patients and healthcare providers (Figure 1). Inspired by a familiar layout of a transit system, the map features color-coded lines representing key aspects of the vitiligo journey: medical treatments, emotional support, and cosmetic solutions. Humor in naming the elements adds a lighthearted touch, making complex processes more approachable.

The map is organized into three main sections:

- Highlands: Healthcare and treatment experiences
- Foggy Bottom: Social support and alternative treatments
- Long Island: Non-treatment and cosmetic management options

This structure helps patients visualize pathways for treatment, alternative options, or acceptance and coping strategies.

#### 2.3.2 Comprehensive approach

VPJM uniquely includes both treatment and non-treatment pathways, validating diverse patient choices. Each “station” represents a critical interaction—whether with the healthcare system, community, or a “moment of truth” where expectations meet reality.

The journey begins at “White Spot,” symbolizing initial symptom awareness. The treatment pathway tracks progression from early online research (“Dr. Google”) to consultations and various therapies. Non-treatment paths include stations like “Beautiful,” a hub for cosmetic solutions and social support, highlighting non-medical coping as a valid choice (8).

#### 2.3.3 Timestamps and patient expectations

A standout feature of the map is its timestamps, which:

• Set realistic timelines for treatment progress
• Emphasize adherence by showing timeframes for visible improvements and relapse risks

The cyclical nature of vitiligo management is illustrated by a “Maintenance Circle,” stressing ongoing care to prevent relapses (9). This is vital, as over 40% of patients relapse within 4 years of achieving repigmentation.

#### 2.3.4 Key insights and special features

Several special features provide deeper insights into the vitiligo experience:

1. Vicious Circle: Represents the common year-long cycle of self-diagnosis, self-management, and alternative treatments before seeking expert care.
2. Lottery Line: Acknowledges rare cases where dietary changes significantly impact vitiligo, highlighting nutrition's role and occasional misdiagnoses (10).
3. Early Bird Station: Shows patients who discontinue treatment prematurely—often within 7 months—mistakenly believing their symptoms are resolved.

By mapping the multifaceted journey of vitiligo patients, the VPJM offers not only a practical roadmap for care but also a tool for managing expectations and improving communication between patients and providers.

### 2.4 Practical applications

The VPJM holds promise for advancing patient-centered dermatology in the following ways:

a. Clinical Workflows: By providing a structured understanding of the patient journey, the VPJM can improve communication between patients and providers, set realistic treatment expectations, and enhance adherence.
b. Healthcare Provider Training: The map can be used to educate providers about the holistic needs of vitiligo patients, including psychosocial support and interdisciplinary care.
c. Health Policy and Advocacy: Policymakers can leverage the VPJM to design interventions that address gaps in care and promote equitable access to treatment.
d. Chronic Disease Management: While tailored for vitiligo, the VPJM's principles can be adapted to other chronic conditions, offering a blueprint for improving patient journeys in diverse healthcare settings.

### 2.5 Limitations

Developing healthcare tools like the VPJM often prioritizes addressing patients' immediate needs over achieving academic rigor, reliability, or broad generalizability (11). The VPJM is not intended as a comprehensive disease management guide but as a practical, user-friendly resource to support informed decision-making in clinical and support group settings.

This study has several limitations. Data were primarily sourced from an internal database and online surveys, which may limit generalizability. Common issues with online surveys, such as selection bias and restricting participation to internet users, are present. Additionally, English-only documents likely excluded non-English speakers. The interpretation relied on expert opinion, and patient-reported outcomes are inherently subject to recall bias, measurement errors, and potential misinterpretation. Finally, the VPJM has not yet been validated across all stakeholder groups, and further research is needed to evaluate its impact on patient outcomes and satisfaction.

## 3 Conclusions

To our knowledge, the VPJM is the first comprehensive patient journey map for individuals living with vitiligo, encompassing both treatment and no-treatment options. It highlights a non-linear journey that contrasts sharply with traditional patient journey maps, which typically suggest a clear start and endpoint. Instead, the vitiligo patient journey begins well before initial care and extends beyond discharge, characterized by multiple interconnected pathways and recurring loops of treatment and relapse.

The VPJM charts the path from pre-diagnosis through various decision-making points, leading to treatment or non-treatment pathways. Visually represented as a transit map with stations, lines, and estimated travel times, the VPJM reflects the non-linear and cyclical nature of care, including treatment phases, self-care missteps, and relapses.

Our findings reveal that each patient's journey is unique, varying across genders, cultures, and healthcare systems. Patients often engage with diverse providers—from general practitioners to mental health counselors—each offering distinct contributions. Importantly, the VPJM highlights the critical role of community support in decision-making, showing that many patients delay effective treatment due to misinformation or resource gaps. This underscores the need for early consultation and clear communication of treatment options, objectives, and timelines by healthcare providers and support groups. The map also emphasizes the importance of maintenance therapy to prevent or delay relapses.

We envision the VPJM as a practical tool for healthcare and support group settings, sparking meaningful conversations with newly diagnosed or returning patients. Policymakers and health system managers can also leverage the map to identify gaps in care, optimize resources, and improve system efficiencies (12). Such efforts could enhance health literacy, empowering patients to take a more active role in managing their condition.

While this research is a meaningful step toward creating patient-centered journey maps for vitiligo, it has limitations. The analysis relies on a single dataset and self-reported outcomes, underscoring the need for further research across diverse populations and countries. Despite this, the VPJM provides a strong foundation for future collaborations to deepen our understanding of the complex factors shaping the vitiligo experience.
